# Using Discrete-Event Simulation to Model Web-Based Crisis Counseling Service Operation: Evaluation Study

**DOI:** 10.2196/46823

**Published:** 2024-08-07

**Authors:** Byron Chiang, Yik Wa Law, Paul Siu Fai Yip

**Affiliations:** 1 Centre of Suicide Research and Prevention University of Hong Kong Hong Kong China (Hong Kong); 2 Department of Social Work and Social Administration The University of Hong Kong Hong Kong China (Hong Kong)

**Keywords:** discrete-event simulation, community operational research, queuing, web-based counseling, service management, repeat users

## Abstract

**Background:**

According to the Organisation for Economic Co-operation and Development, its member states experienced worsening mental health during the COVID-19 pandemic, leading to an increase of 60% to 1000% in digital counseling access. Hong Kong, too, witnessed a surge in demand for crisis intervention services during the pandemic, attracting both nonrepeat and repeat service users during the process. As a result of the continuing demand, platforms offering short-term emotional support are facing an efficiency challenge in managing caller responses.

**Objective:**

This aim of this paper was to assess the queuing performance of a 24-hour text-based web-based crisis counseling platform using a Python-based discrete-event simulation (DES) model. The model evaluates the staff combinations needed to meet demand and informs service priority decisions. It is able to account for unbalanced and overlapping shifts, unequal simultaneous serving capacities among custom worker types, time-dependent user arrivals, and the influence of user type (nonrepeat users vs repeat users) and suicide risk on service durations.

**Methods:**

Use and queue statistics by user type and staffing conditions were tabulated from past counseling platform database records. After calculating the data distributions, key parameters were incorporated into the DES model to determine the supply-demand equilibrium and identify potential service bottlenecks. An unobserved-components time-series model was fitted to make 30-day forecasts of the arrival rate, with the results piped back to the DES model to estimate the number of workers needed to staff each work shift, as well as the number of repeat service users encountered during a service operation.

**Results:**

The results showed a marked increase (from 3401/9202, 36.96% to 5042/9199, 54.81%) in the overall conversion rate after the strategic deployment of human resources according to the values set in the simulations, with an 85% chance of queuing users receiving counseling service within 10 minutes and releasing an extra 39.57% (3631/9175) capacity to serve nonrepeat users at potential risk.

**Conclusions:**

By exploiting scientifically informed data models with DES, nonprofit web-based counseling platforms, even those with limited resources, can optimize service capacity strategically to manage service bottlenecks and increase service uptake.

## Introduction

### Background

According to the Organisation for Economic Co-operation and Development, digital counseling access across its member states increased by 60% to 1000% during the COVID-19 pandemic [1]. These figures are comparable to use statistics from Hong Kong’s only 24-hour youth-based crisis intervention service, Online Crisis Support for Youth or Open Up [2], which served 259% more cases in November 2020 than in November 2018. Wong et al [3,4] showed that the significant negative effects of population-level health crises and social unrest on young people’s mental health led to sudden and unpredictable service demand peaks. To cope with critical bottlenecks at the service operation level, Open Up’s management increased service capacity with new hires and ramped up marketing campaigns to encourage help-seeking behavior, only to discover later that the adjustments had a negligible impact on the service uptake rate and failed to ameliorate working conditions for Open Up employees. The service thus failed in its ultimate aim of optimizing operational efficiency based on the evidence-informed resource estimates that such digital counseling platforms generally seek to achieve.

In such situations, operations researchers apply queuing theory to optimize for-profit call center service operations [5]. In health care, steady-state assumptions [6] and service mechanisms such as priority queues and admission cutoffs are summoned to address capacity bottlenecks in emergency departments and critical care units [7,8]. However, documented applications in community-based emotional support services for people with potential mental health comorbidities are lacking. The slow adoption of such applications can be partly attributed to the complex mathematics of queuing theory and partly to unrealistic steady-state assumptions that contradict the standard of care in the crisis hotline context. Hence, there is a need to tackle the digital social care problem using discrete-event simulation (DES), a tool that applies key concepts of queuing theory to solve complex supply-demand problems. DES has a relatively low entry barrier for social service managers. It casts light on operation bottlenecks, allowing users to avoid decisions likely to undermine crisis service operations. Managers also find the component of DES pertaining to capacity upper and lower bound estimates useful for budgeting purposes.

### Objectives

This paper lays down the theoretical groundwork needed for DES and reports the first-ever application of DES in crisis service operations to evaluate the service capacity-demand gap, an application with significant implications for service development. The first part outlines the working conditions faced by the Open Up operations team during 2019-2020 and the models that can be applied to simulate these conditions, and the second part comprises an analysis section exploring different scenarios by which the operations team can better serve users. The proportion of repeat users in service operation is also estimated using DES.

## Methods

### Overview of Crisis Service Operating Conditions

#### Incoming Traffic and Response Capacity

Between October 2018 and November 2020, a total of 142,722 visitors reached out to the service. Of these 142,722 users, 39,137 (27.42%) were “just visiting” without accepting the terms of service (TOS). Of the 103,585 users who accepted the TOS, counselors managed to convert 60,079 (42.1%) chat requests into actual cases, leaving 43,506 (30.48%) to drop out before case intake. During late evening peak hours, 3 to 5 active web-based counselors were assigned to cope with 13 to 16 chat requests per hour. Demand was lowest during the hours of 5 AM to 9 AM, when 2 active counselors at most were on duty to handle incoming cases.

TOS uptake is independent of time at hourly and monthly intervals. Hence, TOS acceptance for a sequence of users 1,..., *n* is a Bernoulli process *B_n_* [9]. This discrete stochastic process takes 2 values, coded as either 0 or 1. As the probability of acceptance over this period is *P*=*.*73,

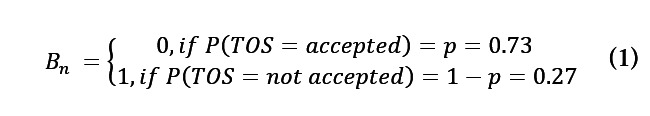



By decomposition, the rate or intensity *λ* at time *t* is given by

*λ*(*t*,*TOS* = *accepted*) = *pλ*(*t*)

*λ*(*t*,*TOS* = not *accepted*) = (1 − *p*)*λ*(*t*) **(2)**

#### Users

Two types of users are served by Open Up: *repeat* users and *nonrepeat* users. During the period under study, a disproportionate amount of time and resources was allocated to serving repeat users [10,11], a group of frequent visitors classified as highly agitated with mental health issues who pose a challenge to unexperienced counselors in service operation [12]. By contrast, nonrepeat users were new to Open Up. The exact breakdown by incoming case and user type is shown in Table 1. Since its inception, the service has attracted a loyal following of users who rely on the digital on-demand counseling platform for regular mental health upkeep. Although service abuse is rare, counselors may choose not to respond to additional chat requests from users who launch multiple concurrent chats from the same IP address. If such abuse persists, the abuser’s right to service may be suspended for 24 hours.

**Table 1:** Breakdown of incoming service users, from December 1, 2019, to November 30, 2020.

			Users, n (%)
**TOS^a^ status (n=83,013)**			
	Accepted	62,081 (74.78)	
	Not accepted	20,932 (25.22)	
**User type (n=62,081)**			
	Nonrepeat	44,125 (71.08)	
	Repeat	17,956 (28.92)	
**Cases served (upon TOS acceptance; n=62,081)**			
	Valid	35,055 (56.47)	
	Invalid	27,026 (43.53)	
**Valid cases by user type (upon TOS acceptance; n=62,081)**			
	Nonrepeat	27,249 (77.73)	
	Repeat	7806 (22.27)	

^a^TOS: terms of service.

User interarrival times *τ_a_*, that is, the difference in arrival times between users *n* and *n*+1, are not stationary by hour. Neither the standard Erlang-C (M/M/N) system based on N servers and an infinitely lengthened queue nor the Erlang-A (M/M/N+M) model that additionally assumes queue abandonment after an exponential time, along with assumptions of steady-state arrivals and exponential service duration, can provide reasonable capacity estimates [5,13]. However, for nonrepeat arrivals, the random variable follows the independent increment property of the Poisson counting process, that is, no 2 chat requests from the same *identifier*, such as an IP address, telephone number, or instant messaging account ID, can be initiated around the same time. Therefore, the sequence of user arrivals can be modeled as a nonhomogeneous Poisson process. The memoryless property is provided by the interarrival time *τ_a_*.

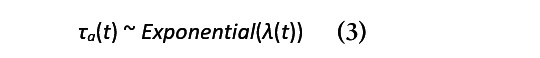



with

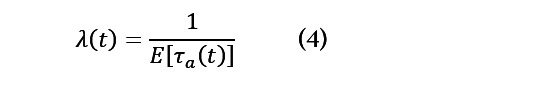



The conditional intensity *λ*(*t*) in equation 3 is the arrival rate per *unit of time*, which is expressed as a function of *t* epochs. By definition of the exponential distribution, *λ*(*t*) is the inverse of the aggregated mean interarrival time *E*[*τ_a_*(*t*)].

Ibrahim et al [5] identified 8 key features of call center arrival processes that can be applied in the context of crisis hotlines. They boil down to 4 main statistical properties: seasonality, overdispersion, autoregressive effects, and exogenous factors. Hence, stochastic time-series forecasting models can be applied to the normalized variable *BoxCox*(*E*[*τ_a_*(*t*)]) to predict future arrival rates from which future capacities can be estimated. The expected bias-adjusted arrival rates at a 95% CI can be piped back into the DES to find the lower and upper bounds of the web-based counselors needed to fill each shift.

In the simulation, a version of the unobserved components model (UCM) with Kalman smoothing [14] is specified to additively decompose *BoxCox*(*E*[*τ_a_*(*t*)]) into a local level *µ*, a time-of-day-varying frequency-domain seasonal component *γ*, a combination of dummy marketing campaign regressors *h*, and a first-order autoregressive irregular *ε*. As time-series regression analysis suggested no marked difference in use patterns between regular workdays and public holidays, holiday effects are excluded from *h*. This observation may be attributable to COVID-19 pandemic–related responses such as school suspensions and work-from-home arrangements, which created a fine line between holidays and regular workdays. The full UCM specification is as follows:

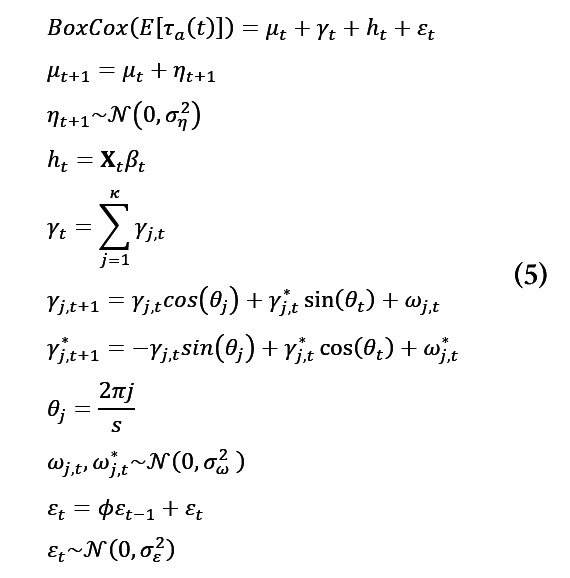



In equation 5, *γ* is specified parsimoniously as a truncated series, which becomes deterministic when *ω_j,t_* = 0 and ω^*^_j,t_ = 0. The optimal cutoff *κ*=6 is provided by the model giving the lowest applicable information criterion value, and period *s*=12 corresponds to the data being aggregated into 2-hour intervals.

Repeat arrivals tend to deviate from the independent increment property and have a memory of past events. Such deviation occurs when frequent users anxiously make additional chat requests [15,16] within a short period of time. Formally known as the Hawkes process, this type of user arrival consists of root Poisson events called *immigrants*, which can have self-exciting properties that spawn additional arrivals called *offspring* [17]. The intensity function of this branching structure is given by the following equation:

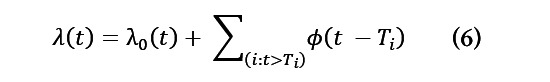



In equation 6, *λ*_0_(*t*) is the time-dependent nonhomogeneous Poisson base intensity for the *immigrants*, derived from equations 3, 4, and 5. *λ*_0_(*t*) is modulated by the memory kernel sum of past events ∑ *ϕ*(*t* − *T_i_*) to generate *offspring* events. Given that counselors serve at most only 1 event per branch, *immigrant births* are the only meaningful arrival events. Their *offspring* can be purged from the total arrival count to restore the independent increment property, meaning that only the base rate function is needed to determine the overall capacity, that is, *λ*(*t*) ≈ *λ*_0_(*t*).

Equation 3 has to be implemented with extra care because the rate function *λ*(*t*) for *t* ∈ (0*,T*) needs to be drawn from the same probability density function (PDF). The 1D thinning algorithm described in the study by Lewis and Shedler [18] is used to generate users from the same PDF on an interval-interval basis. As the authors recommended setting *λ*^∗^(*t*) = *max*(*λ*(*t*)) [18], computation is expected to be slow owing to the large 2-hour timestep intervals specified in equation 5. In addition, note that minor contributions from the Fourier terms and autoregressive components in equation 5 will matter after the bias-adjusted inverse Box-Cox transformation suggested in the study by Hyndman and Athanasopoulos [19] is applied to recover *E*[*λ*(*t*)].

In their modeling of call center arrivals, Ibrahim et al [5] also raised the issue of skillset matching, which has an impact on queue performance as clients wait for suitable counselors to serve them; for instance, on multilingual platforms where counselors are fluent only in the subset of service languages offered, the arrival process can be decomposed into the additional downstream Bernoulli processes (described in the Incoming Traffic and Response Capacity section) to better reflect reality [5]. The relevant dequeuing logic can then be incorporated into the model to simulate actual queuing experiences. The service simulations require no decomposition by language choice because all service members are trilingual.

The validity of the prospective capacity forecasts derived from the bias-adjusted expected arrival rate rests on the assumption that repeat users, if served almost immediately, stop generating *offspring* arrival events.

Despite the lack of suitable alternatives, counting only the first *identifier* instance arriving within a 2-hour time frame may still overestimate the actual demand. In particular, when repeat users anxiously go on a self-excitation binge, service requests launched from different devices by the same user may still pass as nonrepeat cases. Mischaracterizing *offspring* as *immigrant births* can render the *ε* in equation 5 heteroskedastic. The White test can be used to determine whether the *ε* in the UCM model is constant.

#### Shifts

Full-time counselors may assume 1 of 2 roles. *Duty officers* are responsible for overseeing the entire operation during their shift, coaching volunteer counselors during chats, and dealing with users at immediate risk of suicide. *Full-time counselors* are responsible for handling multiple chats concurrently, regardless of the user’s inherent risk. A counselor can alternate between roles as needs arise (eg, “Joe,” a full-time counselor, assumes the duty officer’s role while “Brandon,” the duty officer, takes a meal break). These types of shifts are best laid in parallel with each other.

#### Service Duration

All counselors rate users on an ordinal scale with 4 categories of risk levels and invest their time strategically. Table 2 summarizes the durations of chats, excluding waiting time. The focus is clearly on cases categorized as *high risk* or *crisis*, in which an average of 92.0 (SD=47.3) minutes is spent on carrying out the full crisis intervention routine. By contrast, an average of just 50.2 (SD=31.6) minutes is spent on each case categorized as *low risk*, whereas groups categorized as *medium risk* receive attention for an average of 72.0 (SD=38.9) minutes. After excluding extreme outliers outside the 1.5×IQR, the remaining chat durations were plotted against the theoretical beta distribution. The resulting quantile-quantile plots appear linear, suggesting that chat duration *τ_c_* follows the beta distribution, that is, *τ_c_* ∼ *Beta*(*α_c_,β_c_*), where *α_c_* and *β_c_* are shape parameters in real positive numbers.

**Table 2:** Descriptive summary of chat processing times by risk level and user type, from December 1, 2019, to November 30, 2020.

User type and risk level			Users, n (%)		Values, mean (SD)^a^		Values, median (IQR)^a^
**Nonrepeat (n=27,249)**							
	Low^b^	24,266 (89.05)		50.07 (30.97)		44.80 (45.45)	
	Medium^b^	2815 (10.33)		70.85 (36.80)		67.20 (53.35)	
	High or crisis^b^	168 (0.62)		89.45 (44.18)		82.31 (55.36)	
**Repeat (n=7806)**							
	Low^b^	6932 (88.80)		51.2 (34.49)		43.29 (49.66)	
	Medium^b^	813 (10.42)		75.25 (45.78)		66.99 (66.34)	
	High or crisis^b^	61 (0.78)		121.29 (83.30)		90.29 (82.28)	
**All^c^ (n=35,055)**							
	Low	31,198 (89)		50.21 (31.65)		44.55 (46.31)	
	Medium	3628 (10.35)		71.95 (38.87)		67.18 (55.70)	
	High or crisis	229 (0.65)		92.01 (47.32)		83.18 (58.53)	

^a^Outliers outside the 1*.*5×IQR range are removed.

^b^Mann-Whitney *U* test (nonparametric 2-tailed *t* test); *P*=.10 for low, *P*=.12 for medium, *P*=.14 for high or crisis.

^c^Kruskal-Wallis *H* test (nonparametric ANOVA); *P*<.001.

##### Queue Discipline and Queuing Time

Cases are served on a first-in, first-out basis. Users entering the chat room and accepting the TOS join the queue and wait to be served. If counselors are available, a *zero-wait* case [13] will be dequeued (removed from the queue) immediately after accounting for processing delays caused by network and system latencies. To allow waiting times to follow the exponential distribution, cases picked up within a 60-second window should be treated as *zero-wait* cases.

Users remaining in the queue will either drop out or wait for the next available counselor. The waiting times are key to finding *patience*, the time users spend waiting in the first-in, first-out queue until they drop out; and *virtual waiting time*, the time users with infinite patience will wait to be served [13]. These variables are not observed but can be estimated via survival analysis [13]. The distribution of *patience* is estimated by right-censoring picked-up cases and treating dropout cases as observed events and vice versa for *virtual waiting time*.

As the quantile-quantile plots indicate that *patience τ_r_* is exponential, that is, *τ_r_* ∼ *Exponential*(*_E_*[^1^*τr*]), the survival function is fitted with an exponential model. The estimated mean and SD of *patience* by user type are tabulated in Table 3. Log-rank tests return *P*<.001, suggesting that separate modeling parameters for repeat and nonrepeat users should be assigned in the DES model.

**Table 3:** Patience (the time users will spend waiting in the first-in, first-out queue before they renege) and virtual waiting time (the time users with infinite patience will wait to be served) by user type and estimated with an exponential survival model, from December 1, 2019, to November 30, 2020.

		Values, mean (SD)
**Nonrepeat**		
	Patience^a^	3.4550 (0.0351)
	Virtual waiting time^b^	3.1682 (0.0348)
**Repeat**		
	Patience^a^	5.2895 (0.0765)
	Virtual waiting time^b^	5.0100 (0.0891)
**All**		
	Patience	4.0617 (0.0338)
	Virtual waiting time	3.6759 (0.0343)

^a^Log-rank test; *P*<.001.

^b^Log-rank test; *P*<.001.

It was part of Open Up’s service design during the period under study that the chat system was unable to drop reneged cases automatically and that counselors could not differentiate these cases from cases of users still waiting. We refer to these in-and-out situations as *zombies*, originally a technical term in computer operating systems that describes terminated processes in the process table. Zombies remain in the case queue until assigned to a counselor, who will have to file a counselor postchat survey before the chat can be terminated gracefully.

##### Estimating the Valid Case Count

Service operation defines a case as *valid* when it contains at least 4 conversation exchanges between a user and a counselor. Unfortunately, this count cannot be modeled in the simulation because many latent factors are at play, causing cases to terminate before the cutoff has been reached. Hence, the simulations count only reneged cases as *invalid*; *valid* cases are those picked up by a counselor regardless of the number of exchanges during the conversation. That said, a ballpark figure that approximates the actual *valid* case count is provided by tallying all cases lasting at least 7.5 minutes. This time value is derived from the median of invalid case times and can be used to facilitate capacity discussions with stakeholders.

### Open Up

Open Up is Hong Kong’s first foray into text-based counseling [2]. It can trace back its origins to 2015-2017, when Hong Kongers were left reeling by a cycle of student suicides that claimed 588 young lives. The deaths raised concerns over the mental well-being of 2.16 million individuals aged 10 to 35 years (Hong Kong’s midyear population in 2019 was 7.51 million) [20] and called for a community-based response of crisis intervention in the age of social media; for instance, current service provision has imposed access barriers such as fixed operating hours and maximum age limits [21,22] at community-based youth centers [23], which did not match with youth’s help-seeking behavior. Hence, a free, 24-hour texting service for digital natives to let off steam is anticipated to benefit outliers who will otherwise be left to their own devices. To make it accessible to the public, the text-based service is trilingual in colloquial Cantonese, simplified Chinese, and English. It is also cross-platform compatible, allowing users to seek help on the web portal, WhatsApp, Facebook Messenger, WeChat, and SMS [2].

The service had previously rolled out a rule-based self-reporting chatbot triage system to screen and prioritize cases [2] and had considered upgrading to a heuristics-based natural language processing model [24] to improve the uptake rate. In practice, the chatbot identified a higher-than-anticipated number of false positives. Table 4 presents a breakdown of converted cases by maximum risk levels and screener type. The self-reporting system rated 30.35% (1530/5402) of the cases as having a *high* or *crisis* risk of suicide, contradicting the results of human-screened cases, which identified only 1.04% (573/55,037) of the cases as belonging to these 2 classes of suicide risk. Triage was suspended in November 2019 after spot checks revealed that some users were overstating their actual condition to jump the queue, putting the operating team under significant strain. The trade-off arising from this decision is that zombies are no longer evicted from the queue.

**Table 4:** Breakdown of converted cases by maximum risk level and screener, from October 1, 2018, to November 30, 2020 (n=60,079)^a^.

Maximum risk level	Counselor-screened cases (n=55,037), n (%)	Chatbot-screened cases (n=5042), n (%)
Low	49,083 (89.18)	1863 (36.95)
Medium	5381 (9.78)	1649 (32.71)
High or crisis	573 (1.04)	1530 (30.35)

^a^The chatbot triage system was enabled between October 1, 2018, and November 4, 2019, and momentarily on April 6, 2020. Data from before January 2020, when the first case of COVID-19 was confirmed in Hong Kong, are included to increase the number of chatbot-screened samples.

### DES and Time-Series Solution

A novel hybrid DES and time-series solution was strategically designed and applied to address capacity shortages at Open Up. Tailor-made DES programs are used to simulate a real-world system that consists of multiple discrete events. In this study, discrete events by user type, including user arrivals, simultaneous chats, shift breaks, and the completion of postchat surveys were simulated. Several worker combinations needed to address the immediate supply-demand discrepancy were suggested, although much of the discussion in the Results section relies on the small fine-tunings that such services in densely populated cities can adopt to improve user waiting times. The models can also be applied to various service settings that aim to improve overall operational efficiency without compromising service quality.

### Data

Case data from the service’s Multi-channel Crisis Intervention System database were used to derive all parameters needed for the simulation. The database backend is currently maintained by the IT team of the Hong Kong Federation of Youth Groups. The subset we received consists of time-stamped user chat logs, user identifiers, service state changes, and worker sign-in and sign-out information. Data from December 1, 2019, to November 30, 2020, were used to calculate user patience and generate chat duration statistics. Capacity was evaluated retrospectively using actual interarrival times from November 2020, and the results were compared with forecasts from DES. Time-series modeling and the forecasting of interarrival rates were carried out on a rolling basis from April to August 2020 and from October to November 2020 in 2-hour time steps. Downsampling by piece-wise cubic Hermite polynomial interpolation (ie, interpolating the rate function with a set of Hermite splines) was conducted to speed up the sampling of interarrival rates in DES.

Python libraries were used to perform all statistical analyses needed. *NumPy* (version 1.19.2) [25] and *pandas* (version 1.1.3) [26,27] were used to process the data and generate descriptive statistics, while *SciPy* (version 1.5.2) [28] was used to run chi-square tests comparing the actual data distribution with a theoretical probability distribution, and *lifelines* (version 0.25.10) [29] was used to prepare survival analysis of queuing time. Time-series analysis was conducted using *statsmodels* (version 0.12.1) [30]. The simulation model was specified on the DES library *SimPy* (version 4.0.1) [31] and could be downloaded from the GitHub repository [32]. Visualizations were performed primarily on *seaborn* (version 0.11.0) [33], with Matplotlib (version 3.32) [34] resorted to when necessary.

For evaluations conducted retrospectively, each 30-day service operation simulation was bootstrapped 1500 times using the actual interarrival times from November 2020 to generate an upper bound, lower bound, and average bound. Owing to small rounding errors in full 64-bit double-precision floating-point arithmetic, the cumulative service period was under 30 × 24 × 60 = 43*,*200 minutes by an infinitesimal amount. The Python code handled this slight underflow with a round-robin sequence of interarrivals, which added a few extra cases from the top of the list to cap off the simulation at time point 43,200.

### Ethical Considerations

As principal investigator, PSFY was given permission by the University of Hong Kong Human Research Ethics Committee (HKU HREC; EA1709039) to conduct data collection and analysis on the subject matter. This analysis did not receive exemption from HKU HREC. According to the HKU HREC project application amendment form, no additional review from HKU HREC is needed because this study does not involve a change in the project title, investigator, project period, research method, participant sample, recruitment method, and procedures, as well as supporting documents. The principal investigator has granted other authors of this paper permission to use the data.

## Results

### Retrospective Evaluation of Capacity Based on Actual Interarrival Times

In November 2020, service operation managed to convert 0*.*7506 × 0*.*5072 = 38.07% (3493/9175) of the visitors into actual users. Of the 9175 incoming visitors, 2288 (24.93%) rejected the TOS, implying that any proposed change will be unable to increase the conversion rate beyond 75.06% (6887/9175). As the service operator was interested in maximizing the number of service uses rather than the number of unique users, the simulation based on actual arrival times assumed that all arrivals followed the independent increment property.

### A Base Case Approximating Existing Service Capacity

**Figure 1:**
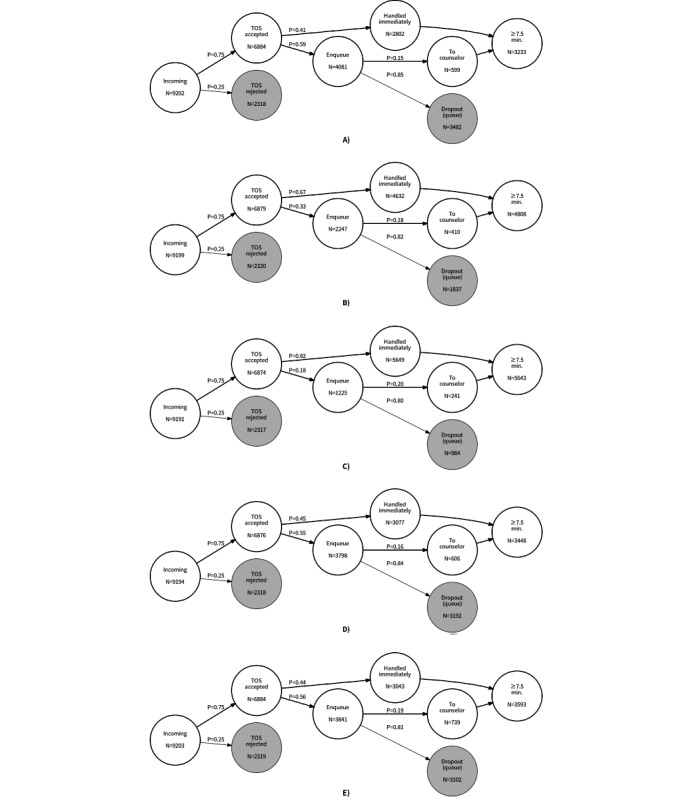
Comparison of different adjustments to Open Up service operation. TOS: terms of service. (A) Base case. (B) Optimum case. (C) Saturation case. (D) Case cutting processing time. (E). Case without zombies.

A base case scenario (Figure 1) was simulated to replicate the actual breakdown in the Retrospective Evaluation of Capacity Based on Actual Interval Times section. A total of 15 counselors operated 4 shifts, with each shift providing a combined shift-wide counseling capacity of at least 8 simultaneous cases. Upon accepting the TOS, 40.7% (2802/6884) of the users were handled immediately, while 59.3% × 14.68% = 8*.*7% (599/6884) of the users were waiting in line to be matched with a counselor. Meanwhile, 59.3% × 8.53% = 50.69% (3482/6884) of the users dropped out without speaking to a counselor. The overall conversion rate was 74.81 × (40.70 + 8.70) = 36.96% (3401/9202), close enough to the 38.07% (3493/9175) actually achieved in November 2020.

### Assessing the Service Pledged With the Base Case

**Figure 2:**
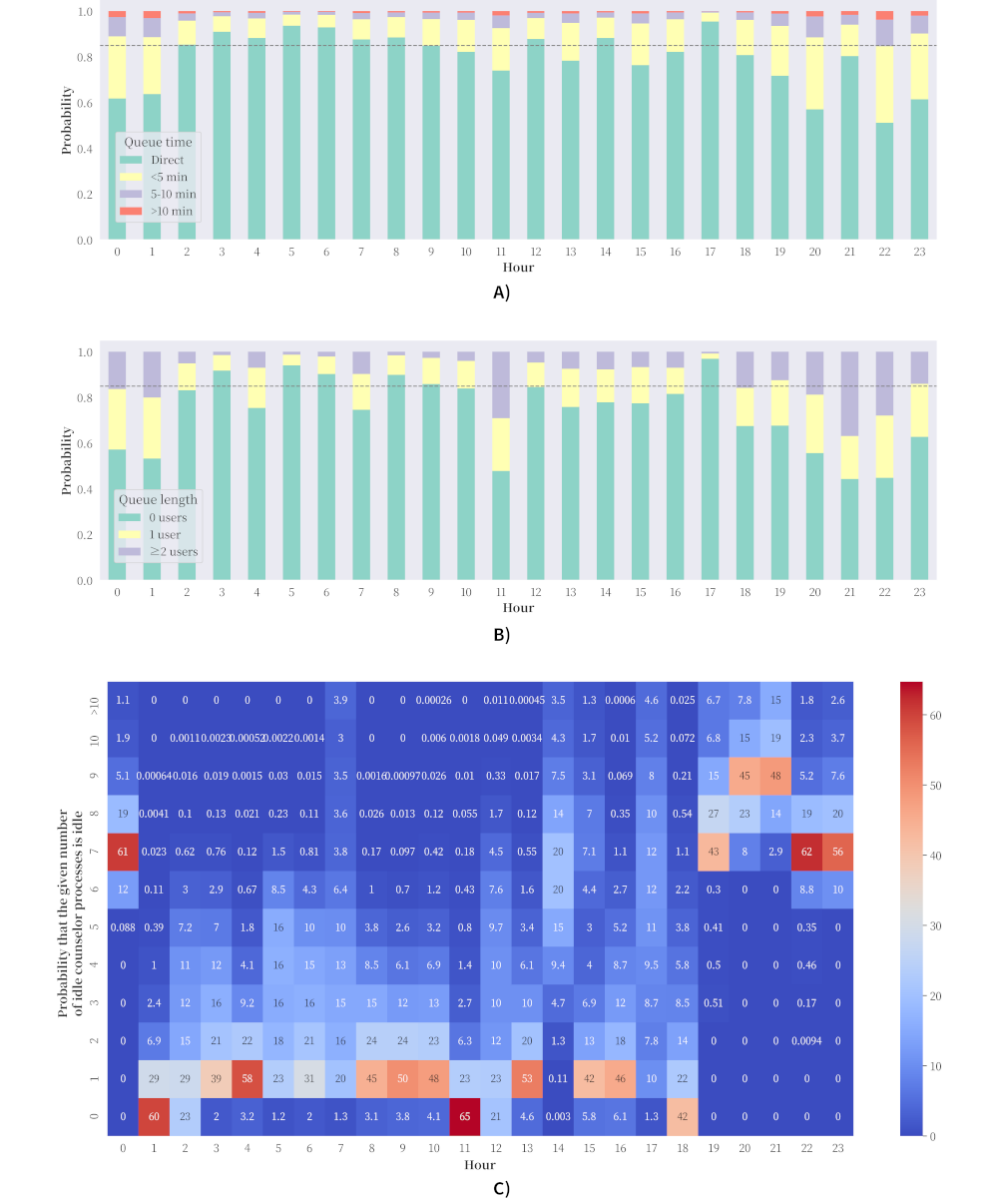
Probability plots for the base case scenario mentioned. (A) Hourly queue length probabilities. (B) Hourly queue time probabilities. For transfer cases, only the first instance is counted in the probability calculations. (C) Probability distribution of hourly service operation counseling capacity.

Figure 2A shows the hourly queuing time probabilities for users who remain in the queue. These sets of plots can be used to conduct conformity assessment of a service pledge. For users who can endure the wait, there is at least an 85% chance that counseling service is no more than 10 minutes away, thus meeting a key commitment in the Open Up service pledge. As indicated in Figure 2B, with the exception of evening and graveyard shift handovers and meal breaks (from 11 AM to noon), there is an 85% chance that no more than 1 person is waiting in line, a second key commitment. Service managers can improve compliance by arranging for or incentivizing more staff to serve during the 11 AM to noon, 7 PM to 10 PM, and midnight to 1 AM time slots. Of note, this user queue is hidden behind the scenes. In practice, the length of the *actual* user queue is often overestimated because it is based on the status of the case queue, which may contain zombies.

### Evaluating the Counseling Vacancy With the Base Case

From a managerial perspective, a counselor should be handling as many cases as are allotted to them because this implies that all counselors are operating at their maximum service capacity. However, a closer examination into counselor vacancy suggests that the anticipated work load should not be measured in such terms. Illustrated in Figure 2C is the probability distribution of unused resource by hour. Counselors are relatively relaxed during shift starts at 2 PM and 5 PM, as well as between 7 PM and midnight when multiple shifts overlap. However, by tabulating all reneged cases that exist in all simulations (N=4,634,407) and expressing those results as a discrete dropout probability density in one hour intervals, the average dropout probability over the 24 hour period was 0.042 (SD 0.033). Meanwhile, the cumulative dropout probability between 2 PM and 5 PM was found to be 10.98% (509,073/4,634,407). However, between 7 PM and midnight the cumulative dropout probability was 45.98% (2,130,854/4,634,407) and the average dropout probability 0.092 (SD=0.024), implying capacity increases should target the evening hours to reduce user dropout, ie increase the number of counselors in different shifts to minimize the difference between the rate at which counselors accept cases and the rate at which users decide to leave the queue.

### An Optimal Scenario and a Capacity Upper Bound

Eleven more worker combinations were simulated to ascertain an optimal scenario that maximizes outcomes at the lowest cost. Time intervals with the highest dropout probability are targeted incrementally using the method outlined in the Evaluating the Counseling Vacancy With the Base Case section.

**Figure 3:**
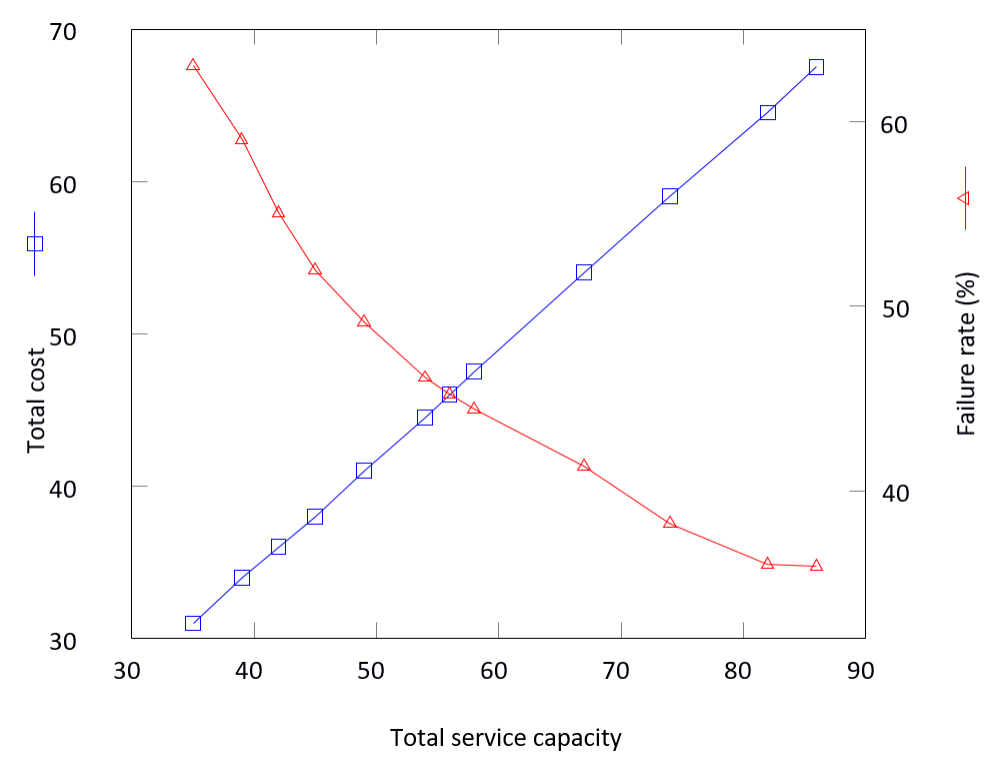
The optimal worker combination is located at the intersection of the cost and effectiveness curves. The use of resources is achieved with greater outcomes.

The optimal worker combination is determined graphically by locating the intersection of the cost and effectiveness curves (Figure 3). *Cost* varies by the hotline operation but, in general, can refer to actual staffing costs, investment in staff and volunteer training, the difficulty of recruiting clinical psychologists or social workers to the platform, and the retention of good volunteers. In our case, an educated guess had to be made because the payroll of full-time hotline staff was delegated to the hiring nongovernmental organizations, and the details were unavailable to us. Upon consideration of relevant factors, the following decimal-based cost values were assigned for Open Up: 1.5 was assigned to each *volunteer* in a shift, 2 to each *full-time counselor*, and 3 to each *duty officer*.

The total cost was tallied for each scenario and plotted against the corresponding total service capacity, given by the total maximum number of chat processes handled during the 4 shifts. The effectiveness or failure rate curve can be produced in a similar manner, only this time with the set of 12 nonconversion rates used in place of total cost.

At the optimum, a daily total of 24 counselors at a total cost of 46 is required to deliver a total service capacity of 56 and an overall conversion rate of 54.8% (5042/9199), a large improvement over the base case conversion rate of 36.95% (3401/9202).

Another point to note is that cost increases linearly, whereas efficiency decreases nonlinearly, meaning that once the capacity upper bound reaches saturation at 64.08% (5890/9191), adding more counselors will have very little effect on the overall conversion rate. Coincidentally, this is also the point at which the minimum service pledge to serve 85% of users in no more than 10 minutes is finally met.

### Strategies to Increase Uptake

#### Overview

It can be difficult to ramp up recruitment at short notice, especially when 60% more staff are needed to reach the optimal capacity level. Hence, our focus now shifts to minimizing workflow bottlenecks. In particular, improvements in the overall conversion rate can be made simply by reducing the average service times and tackling zombie cases. The same worker combination as in the base case is used to facilitate comparisons. Figure 1 presents a cross-comparison with the scenarios discussed in the Results section.

#### Reducing the Average Processing Time

Cases are processed much more slowly than the rate at which users leave the queue. Although little can be done to moderate user impatience, processing times can be reduced to accommodate user behavior. Discussions with seasoned counseling professionals informed by their practice wisdom indicated that 40 minutes is the average length of time needed to service a call requiring basic help. When the beta distribution parameters are scaled accordingly, 4% more cases are handled immediately after TOS acceptance, while the overall conversion rate increases by 3%.

#### Automatic Eviction of Terminated Counseling Cases (Zombies) Still Showing Up in the Queue

Zombie cases drain valuable service resources that could be used to serve other users and that reduce staff self-satisfaction, especially among *volunteer counselors*. Perhaps more importantly, zombies create the false impression that the waiting time is lengthy. As a user’s *position in line* is derived from the case queue, when the queue is filled with zombies, the overstated position in the message prompt may deter users from seeking help.

In 1 sense, removing zombies is a mechanism for managing user expectations and reducing the waiting time in the queue [35]. Assuming that a chat system can be adapted to handle zombies, the overall conversion rate rises by 4.1% when counselors are not required to handle reneged cases, allowing counselors to handle 3.5% more cases immediately and the number of users selected from the queue to increase by 4.5%.

### Prospective Evaluation of Capacity With Time-Series Forecasting to Explore Repeat User Use

**Figure 4:**
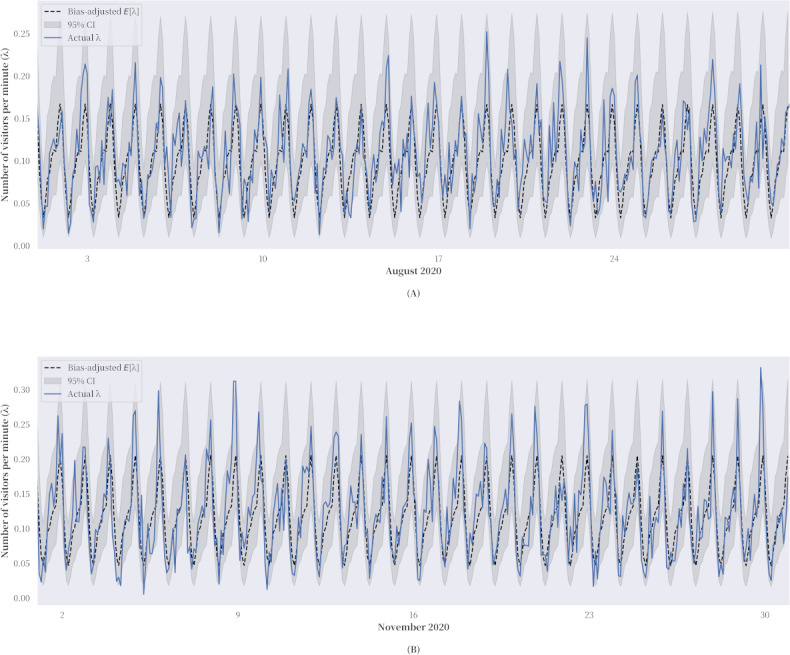
Multistep forecasts versus actual arrival rates in (A) August 2020 (Ljung-Box test: Q=0.17; *P*=.68; White test: H=0.95; *P*=.61) and (B) November 2020 (Ljung-Box test: Q=0.09; *P*=.77; White test: H=1.14; *P*=.16).

A 4-month sliding window was found to produce the best prediction of *λ*, giving the time-series model sufficient flexibility to respond to events in the future. Figure 4 compares the actual arrival rates in August and November 2020 with the multistep forecasts. In both time slices, the bias-adjusted *E*[*λ*(*t*)] closely followed *λ*(*t*) during nonpeak hours, whereas occasional demand spikes during the late night hours in August (ie, between 8 PM and midnight) became more commonplace in November. The White test for the time-series model produced similar results, with the *P* values dropping sharply on data for the 2 months but not significantly enough to suggest that the arrival data are not heteroskedastic. Although some of the demand spikes in the late night hours were initiated by a few well-known repeat users who sought help using another device, much of the demand was due to the service operation’s fair-use policy, which commenced in September 2020 with the aim of helping repeat users to transition from web-based counseling to more in-depth and systematic offline counseling and psychotherapy sessions. In subsequent years, the policy became the Connected Care referral program [36].

The expected total number of user arrivals for November 2020 based on prospective DES was 5544 (95% CI 3177-CI 9388), much lower than the 9175 users actually recorded for this month. Deviations from the actual PDF during thinning or the peaks observed in Figure 4 were the only 2 possibilities to explain the discrepancy. If the expected count is credible, it suggests that repeat users account for 39.57% (3631/9175) of the service operation traffic, which was much higher than the 2.6% to 22% reported in the study by Boness et al [37] and the 28.92% (17,956/62,081) in the official Open Up data but lower than the 60% in the study by Middleton et al [10]. Such a high percentage could be an indicator that the needs of repeat users were not being fully addressed, thus prompting them to launch multiple sessions to locate other counselors who could help them [2]. Furthermore, the promulgation of the National Security Law in Hong Kong on June 30, 2020, triggered a new wave of emigration. We were told that a handful of senior staff had resigned their positions to take advantage of the overseas settlement schemes offered by countries such as Canada and the United Kingdom. It takes time for repeat users to build rapport with new staff.

Optimal capacities can be generated using the method outlined in An Optimal Scenario and a Capacity Upper Bound section.

## Discussion

### Principal Findings

DES was applied to account for time-dependent arrivals, identify system bottlenecks, and evaluate the supply-demand discrepancy of a service desk [5,13]. This was the first time that DES was applied to provide 24/7 services to respond to a crisis at the population level. Open Up’s service niche is underpinned by its philosophy that every minute counts in saving lives. Adhering to this philosophy is not really possible with existing mathematical models that make only a steady-state assumption. Our approach allowed us to incorporate other human factors into the model by optimizing and responding swiftly to ensure timely action. Actual user arrival times were set in the model to produce an expected use number similar to the actual values achieved in the base case scenario. Through DES modeling, we identified areas for unleashing the full potential of the web-based platform under study. From incoming calls to official cases, our simulated model gave an overall conversion rate of 36.95% (3401/9202), which was comparable to the actual values achieved. However, this figure neither met the management team’s expectations nor addressed the population-level demand, both of which can be achieved through capacity optimizations. The overall conversion rate would increase to 54.81% (5042/9199) if the existing staff head count converged with the numbers in the simulations. At saturation, the conversion rate would be capped at 64.08% (5890/9191); further head count increases would have a negligible impact on user uptake but would lead to excessive resource use.

In the base case that achieved a 36.95% (3401/9202) conversion rate, 40.7% (2802/6884) of the cases could be handled directly before capacity adjustments and bottleneck mitigations. We found that 1 of Open Up’s service pledges—“85% chance of a 10-minute waiting time”—was being met. However, its other pledge—“No more than one person waiting in line”—was going unfulfilled during regular staff break cycles (ie, the 11 AM-noon, 7 PM-10 PM, and midnight-1 AM time slots). Capacity mismatch was also found during peak hours (7 PM-midnight time slot), which led to the doubling of the dropout probability (ie, from an average of 5% to >10%). When these holes were plugged, the conversion rate improved, rising to 54.81% (5042/9199). Aware that financial resources are always limited for nonprofit service operations, this study has succeeded in demonstrating a scientific and repeatable modeling approach for tackling system bottlenecks. Managers can deploy strategic solutions to increase system uptake, for example, standardizing the average processing time to 40 minutes, which would increase the overall conversion rate by 3%; and triaging repeat users to a tailor-made care program, which would release an extra 39.57% (3631/9175) capacity to serve nonrepeat users at potential risk. Thus, the study has found profound implications for service development and delivery.

The capacity forecast pipeline used in the study applied a random stochastic point process called the nonhomogeneous Poisson process to model the de-excited sequence of user arrivals as a function of time. A time-series model was fitted to model this time-varying arrival rate, and the results were fed into a DES model to come up with a shift capacity that would minimize the supply-demand discrepancy. For this forecasting approach to work, an assumption needs to be made: repeat users will not return shortly after completing their counseling sessions. Skogevall et al [12] argued that repeat users can be deterred from making frequent calls if their needs are fully addressed by a group of coordinated professionals. A middle ground could perhaps be to target nonrepeat users during peak demand periods. Repeat caller traffic could be redirected to the Connected Care program, with the curfew lifted during nonpeak hours.

Managing the expectations of repeat users is the key to managing the supply-demand discrepancy. The pipeline can be adapted to predict the branching patterns of individual repeat users if the arrival process is specified as a nonhomogeneous Hawkes process, another stochastic process that builds on the memory of the last random arrival event. We postulate that the self-excitation and relapse patterns of these users at different time periods reflect the state of their mental health comorbidities. The successful forecasting of these long- and short-term stress cycles is a potential area for future research, allowing service providers to devise a more holistic package of connected care for repeat users. Engaging them before a mental wellness decline would preempt their service use and prevent them from making too many concurrent calls.

Repeat users who may require intensive, tailor-made care [10,11] are often found to dominate the crisis services [12], which are designed primarily to provide momentary relief to those in emotional distress. Modeling by DES allowed us to pinpoint the surplus capacity (ie, 3631/9175, 39.57%) that could be redirected to nonrepeat users with service needs more closely aligned to the original service mission. However, a valid mechanism for screening users’ risk level needs to be in place to triage them to the Connected Care program. Such a screening mechanism would serve as a safeguarding measure to support both repeat users and nonrepeat users, allowing them to be served in timely fashion by this web-based platform.

Although the simulation described herein was able to provide data-driven insights with little mathematical rigor, it was not sufficiently user-friendly to be used as a stand-alone application. A fruitful direction for future research would be to enhance the user-friendliness of the simulation by integrating it with a graphic user interface, allowing staff to tweak workflows and perform due diligence on staffing requirements before enacting them.

### Conclusions

DES is a systemic approach to modeling and strategically managing service desk operations. The model’s data-driven simulations can be used to forecast and evaluate the resource use, service quality, and uptake rate of crisis services, allowing the population’s acute demand for mental health services to be evaluated retrospectively and forecast prospectively. The viability of DES as a management tool for optimizing service capacity according to user demand has been demonstrated. Had the time-delayed data been available in real time, DES could have been applied concurrently on the entire service operation, allowing managers to fine-tune their live operations to meet changing demand.

Nonprofit services rarely deploy scientific data-modeling methods such as DES to monitor large-scale operations. This study bridges the gap by empowering social agencies to deal with a long-standing challenge: providing last-minute help to a population at risk. Therefore, providers of counseling services of any scale are recommended to embrace and incorporate data-driven approaches such as DES into their decision-making process.

## Abbreviations

DES: discrete-event simulation
HKU HREC: University of Hong Kong Human Research Ethics Committee
PDF: probability density function
TOS: terms of service
UCM: unobserved components model

## Data Availability

The Multi-channel Crisis Intervention System (MCCIS) data set analyzed during this study are not publicly available due to data confidentiality and subject protection, but are available from the corresponding author on reasonable request. The governing body of Open Up shall have the final discretion to honor or deny data access requests to the MCCIS database. The source code for the DES model specification and simulation, the Jupyter Notebook file that cleans the raw data set from the MCCIS database and transforms it into data for further analysis, and the actual interarrival data for November 2020 generated during and analyzed during the study are available in the GitHub repository [32].
